# Preoperative Gamma-Glutamyltransferase-to-Lymphocyte Ratio as an Independent Prognostic Biomarker in Patients Undergoing Radical Cystectomy for Bladder Cancer

**DOI:** 10.3390/medicina62020343

**Published:** 2026-02-08

**Authors:** Tomohiro Matsuo, Shintaro Mori, Hiroyuki Honda, Shota Kakita, Kyohei Araki, Kensuke Mitsunari, Kojiro Ohba, Yasushi Mochizuki, Ryoichi Imamura

**Affiliations:** Department of Urology, Nagasaki University Graduate School of Biomedical Sciences, Nagasaki 852-8501, Japan; s-mori@nagasaki-u.ac.jp (S.M.); h.honda2306@nagasaki-u.ac.jp (H.H.); s-kakita@nagasaki-u.ac.jp (S.K.); araki.k@nagasaki-u.ac.jp (K.A.); kmitsunari@nagasaki-u.ac.jp (K.M.); ohba-k@nagasaki-u.ac.jp (K.O.); mochi@nagasaki-u.ac.jp (Y.M.); ryo-imamura@nagasaki-u.ac.jp (R.I.)

**Keywords:** bladder cancer, radical cystectomy, prognostic biomarker, gamma-glutamyltransferase-to-lymphocyte ratio

## Abstract

*Background and Objectives*: Gamma-glutamyltransferase-to-lymphocyte ratio (GLR) is a prognostic biomarker reflecting oxidative stress and host immune status. However, its prognostic value in patients with bladder cancer undergoing radical cystectomy (RC) remains unclear. This study aimed to investigate whether preoperative GLR predicts survival outcomes following RC. *Materials and Methods*: We retrospectively reviewed 110 patients with urothelial carcinoma of the bladder (pure urothelial carcinoma or urothelial carcinoma with variant histology) who underwent RC at a single tertiary center between 2008 and 2022. GLR was calculated as serum gamma-glutamyltransferase (U/L) divided by absolute lymphocyte count (×10^9^/L) using routine preoperative blood samples. Patients were categorized into low-GLR (≤17.0; *n* = 54) and high-GLR (>17.0; *n* = 56) groups based on the cohort median cut-off (17.0). Overall survival (OS), recurrence-free survival (RFS), and cancer-specific survival (CSS) were assessed using Kaplan–Meier analysis and compared by log-rank tests. Cox proportional hazards models were used, including a preoperative model (Model 1) and a pathology-adjusted model incorporating postoperative variables (Model 2). *Results*: High GLR was associated with significantly worse OS, RFS, and CSS (log-rank: *p* = 0.020, *p* = 0.043, and *p* = 0.003, respectively). In multivariate analyses, high GLR was independently associated with inferior outcomes in both models. In Model 2, high GLR predicted worse OS (hazard ratio [HR] = 2.38; 95% confidence interval [CI] = 1.32–4.28; *p* = 0.003), RFS (HR = 2.37; 95% CI = 1.13–4.99; *p* = 0.020), and CSS (HR = 3.45; 95% CI = 1.56–8.52; *p* = 0.001). *Conclusions*: Preoperative GLR is a simple, inexpensive biomarker independently associated with survival after RC for bladder cancer, even after adjustment for established clinicopathological and pathological factors. GLR may support risk stratification and postoperative management, warranting prospective multicenter validation.

## 1. Introduction

Bladder cancer is a common malignancy of the urinary tract, and radical cystectomy (RC) with pelvic lymph node dissection remains the standard curative treatment for muscle-invasive bladder cancer and selected cases of high-risk non-muscle-invasive disease [1,2]. Despite advances in perioperative management and systemic therapy, oncological outcomes after RC remain heterogeneous, and a substantial proportion of patients experience disease recurrence and cancer-related mortality [1]. Accordingly, improved preoperative risk stratification is needed to support individualized postoperative surveillance and treatment strategies.

Recently, increasing attention has been paid to systemic inflammation- and nutrition-related biomarkers derived from routine blood tests as prognostic indicators in urothelial carcinoma. Several indices, such as the neutrophil-to-lymphocyte ratio (NLR), platelet-to-lymphocyte ratio (PLR), lymphocyte-to-monocyte ratio (LMR), systemic immune-inflammation index (SII), and albumin–globulin ratio (AGR), correlate with oncological outcomes in patients undergoing RC for bladder cancer [1,3,4]. These markers reflect the complex interplay between the host inflammatory response, immune status, and tumor progression, and have been proposed as inexpensive and readily obtainable prognostic tools in daily practice [3,4].

Serum gamma-glutamyltransferase (GGT) is a membrane-bound enzyme involved in glutathione metabolism and oxidative stress regulation. Beyond its traditional role as a marker of hepatobiliary disease and alcohol consumption, accumulating evidence indicates that GGT is implicated in tumor progression, drug resistance, and redox-related carcinogenic pathways in various malignancies [5,6]. Clinically, elevated preoperative serum GGT is an independent predictor of poor survival in patients undergoing RC for bladder cancer, suggesting that GGT-based indices may have prognostic utility in this setting [7]. Given that GGT reflects redox-related metabolism and lymphocyte count reflects host immune status, combining these parameters into a single index may provide complementary prognostic information.

Recently, the gamma-glutamyltransferase-to-lymphocyte ratio (GLR), which integrates serum gamma-glutamyltransferase and the absolute lymphocyte count, has been proposed as a composite biomarker reflecting both oxidative stress–related metabolism and host immune status. An elevated GLR is associated with adverse oncological outcomes in patients with hepatocellular carcinoma following curative surgical treatment, supporting its utility for postoperative risk stratification [8,9]. However, the prognostic significance of preoperative GLR in patients undergoing radical cystectomy for bladder cancer has not yet been clarified.

Our study aimed to investigate the prognostic significance of preoperative GLR in patients treated with RC for bladder cancer at a single tertiary care center. We hypothesized that a high preoperative GLR is associated with worse overall, recurrence-free, and cancer-specific survival, independent of established clinicopathological prognostic factors.

## 2. Materials and Methods

### 2.1. Study Design and Patient Population

This retrospective cohort study included patients who underwent RC for bladder cancer at Nagasaki University Hospital between January 2008 and December 2022. Eligible patients had histologically confirmed urothelial carcinoma (pure urothelial carcinoma or urothelial carcinoma with variant histology) of the bladder and were treated with curative intent. Patients were excluded if they had any active malignancy or a prior history of malignancy (including upper tract urothelial carcinoma), distant metastasis at the time of surgery, a history of chronic hepatitis, heavy alcohol consumption, or incomplete clinicopathological or laboratory data.

The study protocol was approved by the Institutional Review Board of Nagasaki University Hospital (approval no. 12052899) and was conducted in accordance with the principles of the Declaration of Helsinki. Given the retrospective design and the use of routinely collected clinical data, the requirement for written informed consent was waived, and patients were offered the opportunity to opt out via a public notice on the hospital website.

### 2.2. Treatment and Follow-Up

All patients underwent RC with pelvic lymph node dissection according to institutional standards. Given that minimally invasive/robot-assisted RC was introduced during the study period, the surgical approach (open versus laparoscopic/robot-assisted) was determined by the treatment era and institutional availability. The type of urinary diversion was selected by the operating surgeons based on tumor characteristics and patient factors. Neoadjuvant and/or adjuvant systemic chemotherapy was administered at the discretion of the treating physicians in accordance with contemporary guidelines, renal function, and performance status.

After RC, patients were followed according to institutional practice. Follow-up generally consisted of physical examination, routine blood tests, and cross-sectional imaging, including computed tomography of the chest, abdomen, and pelvis, at regular intervals (every 3–6 months for the first 4–5 years and annually thereafter, or as clinically indicated). Disease recurrence was determined based on radiological findings and was confirmed by histopathology whenever feasible.

### 2.3. Data Collection and Definition of GLR

Clinicopathological data were extracted from electronic medical records, including age, sex, body mass index (BMI), smoking history, Eastern Cooperative Oncology Group performance status (ECOG-PS), comorbidities, clinical T stage and clinical nodal status (cN), pathological T stage, concomitant carcinoma in situ, histopathological classification, lymphovascular invasion (LVI), tumor grade, surgical approach, type of urinary diversion, and receipt of neoadjuvant and/or adjuvant chemotherapy.

Preoperative laboratory data were obtained from routine blood tests performed during the preoperative assessment, typically within 2–4 weeks before surgery, while patients were clinically stable and without evidence of infection or acute inflammatory disease. Serum GGT (U/L) and absolute lymphocyte count (×10^9^/L) were recorded. GLR was calculated as GGT divided by the absolute lymphocyte count. For this exploratory analysis, patients were stratified into low- and high-GLR groups according to the cohort median GLR value (cut-off, 17.0).

### 2.4. Outcome Measures

The primary endpoint was overall survival (OS), defined as the interval from the date of RC to death from any cause. The secondary endpoint was recurrence-free survival (RFS), defined as the interval from the date of RC to the first documented local recurrence, regional or distant metastasis, or death from bladder cancer. The third endpoint was cancer-specific survival (CSS), defined as the interval from the date of RC to death from bladder cancer, with deaths from other causes censored. Patients without events were censored at the date of last follow-up.

### 2.5. Statistical Analysis

Continuous variables are presented as median [interquartile range, IQR], and categorical variables as numbers and percentages. Differences in clinicopathological characteristics between the low- and high-GLR groups were assessed using the chi-square test or Fisher’s exact test, as appropriate, for categorical variables and the Mann–Whitney U-test for continuous variables.

Survival curves for OS, RFS, and CSS were estimated using the Kaplan–Meier method and compared between groups using the log-rank test. Median survival times with 95% confidence intervals (CIs) were estimated from the Kaplan–Meier curves when applicable.

Univariate and multivariate Cox proportional hazards regression analyses were performed to identify prognostic factors for OS, RFS, and CSS. Clinical and pathological T stage were dichotomized as ≤T2 versus ≥T3 to reflect the clinically relevant distinction between organ-confined and extravesical disease and to preserve model parsimony given the modest number of events. Variables with *p* < 0.05 in univariate analyses and prespecified clinically relevant covariates were considered for multivariate analyses. To evaluate the prognostic value of GLR in different clinical settings, two multivariable models were constructed: a preoperative model (Model 1) including only variables available before surgery (such as age, sex, ECOG-PS, clinical T stage, cN, and receipt of neoadjuvant chemotherapy), and a pathology-adjusted model (Model 2) additionally incorporating postoperative pathological variables (such as pathological T stage and LVI). Because the GLR cut-off was derived from the cohort median (17.0) for this exploratory analysis, we also conducted sensitivity analyses treating GLR as a continuous variable after natural-log transformation (ln[GLR]) and assessing robustness after additional adjustment for NLR and surgery year (Supplementary Materials: Tables S1–S4). The proportional hazards assumption was assessed using Schoenfeld residuals for the final OS model; no evidence of violation was observed (global test *p* = 0.67). Hazard ratios (HRs) with 95% CIs were reported. Baseline comparisons between groups are presented descriptively and were not adjusted for multiple testing. All tests were two-sided, and *p* < 0.05 was considered statistically significant. Statistical analyses were performed using EZR (Saitama Medical Center, Jichi Medical University, Saitama, Japan), a graphical user interface for R version 4.3.1 (R Foundation for Statistical Computing, Vienna, Austria). No formal a priori sample size calculation was performed because this was a retrospective study including all eligible patients during the study period.

## 3. Results

### 3.1. Patient Characteristics

A total of 110 patients who underwent RC for bladder cancer were included in this analysis. Patients were stratified into a low-GLR group (GLR ≤ 17.0; *n* = 54) and a high-GLR group (GLR > 17.0; *n* = 56) according to the predefined cut-off. The median GLR in the overall cohort was 17.0 [IQR, 12.4–26.1] (Table 1).

The median age at surgery was 72 years [IQR, 65–77], with 85 patients (77.3%) being male. The median BMI was 23.9 kg/m^2^ [IQR, 20.6–25.0]; 61 patients (55.5%) had a smoking history; and ECOG performance status was ≤2 in 94 patients (85.5%) (Table 1).

Regarding clinical stage, cT3 and cT4 disease were observed in 39 (35.5%) and 13 (11.8%) patients, respectively (cT3–4: 52/110, 47.3%), and clinical nodal metastasis (cN+) was present in 9 patients (8.2%). Pathologically, pT3 and pT4 disease were identified in 30 (27.2%) and 11 (10.0%) patients, respectively (Table 1).

Overall, baseline characteristics were largely comparable between the two groups (Table 1). A notable difference was histopathology: the proportion of urothelial carcinoma with variant histology was higher in the high-GLR group than in the low-GLR group (39.3% vs. 20.4%, *p* = 0.038). As expected, preoperative GGT levels (38.0 vs. 19.5 U/L, *p* < 0.001) and GLR (32.5 vs. 14.5, *p* < 0.001) were higher in the high-GLR group, whereas lymphocyte counts were lower (1.39 vs. 1.79 × 10^9^/L, *p* = 0.036). The high-GLR group also had a higher NLR (3.18 vs. 2.25, *p* = 0.036).

### 3.2. Oncological Outcomes According to GLR

**Figure 1:**
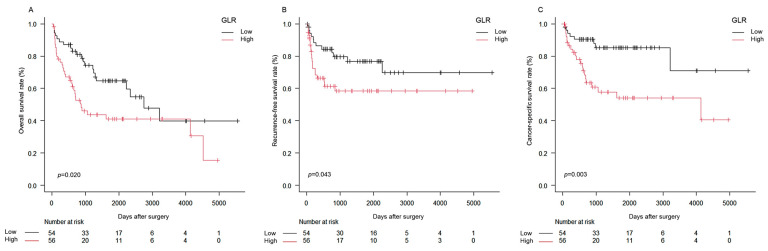
Kaplan–Meier survival curves according to the preoperative GLR. Patients were stratified into low-GLR (≤17.0; *n* = 54) and high-GLR (>17.0; *n* = 56) groups. (**A**) OS, (**B**) RFS, and (**C**) CSS. The high-GLR group showed significantly poorer OS, RFS, and CSS than the low-GLR group (log-rank: OS, *p* = 0.020; RFS, *p* = 0.043; CSS, *p* = 0.003). Abbreviations: GLR, gamma-glutamyltransferase-to-lymphocyte ratio; OS, overall survival; RFS, recurrence-free survival; CSS, cancer-specific survival.

Kaplan–Meier analyses demonstrated that patients in the high-GLR group had significantly poorer OS, RFS, and CSS than those in the low-GLR group (log-rank: OS, *p* = 0.020; RFS, *p* = 0.043; CSS, *p* = 0.003) (Figure 1A–C). The median OS was 2754 days (95% CI = 1332–not reached) in the low-GLR group and 881 days (95% CI = 598–4519) in the high-GLR group (Figure 1A). Although the median RFS was not reached in either group, the RFS curve was significantly worse in the high-GLR group (95% CI: low-GLR, 2263–not reached; high-GLR, 524–not reached) (Figure 1B). For CSS, the median survival time was not reached in the low-GLR group (95% CI = 3217–not reached), whereas it was 4133 days (95% CI = 693–not reached) in the high-GLR group (Figure 1C).

### 3.3. Univariate and Multivariate Analyses

The results of univariate and multivariate Cox proportional hazards regression analyses for OS, RFS, and CSS are summarized in Table 2 (preoperative model; Model 1) and Table 3 (postoperative/pathology-adjusted model; Model 2). In addition, sensitivity analyses using ln(GLR) and NLR as continuous variables, as well as models additionally adjusted for surgery year to address potential temporal bias, are provided in Supplementary Materials Tables S1–S4. 

#### 3.3.1. Preoperative Model (Model 1) (Table 2)

In univariate analyses, high GLR was significantly associated with worse OS (HR = 1.91, 95% CI = 1.10–3.32; *p* = 0.022), RFS (HR = 2.07, 95% CI = 1.01–4.24; *p* = 0.047), and CSS (HR = 3.19, 95% CI = 1.41–7.24; *p* = 0.005). In multivariate analyses, high GLR remained independently associated with poorer outcomes across all endpoints: OS (HR = 2.23, 95% CI = 1.25–3.98; *p* = 0.006), RFS (HR = 2.02, 95% CI = 1.01–4.16; *p* = 0.045), and CSS (HR = 3.31, 95% CI = 1.43–7.66; *p* = 0.003). In this model, age was independently associated with OS (HR = 1.05, 95% CI = 1.01–1.09; *p* = 0.006). Smoking history and advanced clinical T stage were independent predictors of RFS (smoking: HR = 2.27, 95% CI = 1.12–4.61; *p* = 0.024; clinical T stage: HR = 2.00, 95% CI = 1.01–4.08; *p* = 0.048), and clinical T stage remained independently associated with CSS (HR = 2.54, 95% CI = 1.12–5.76; *p* = 0.021).

#### 3.3.2. Postoperative/Pathology-Adjusted Model (Model 2) (Table 3)

In univariate analyses, advanced pathological T stage and LVI were strongly associated with OS, RFS, and CSS, and high GLR also remained significantly associated with poorer outcomes. In multivariate analyses adjusting for pathological variables, high GLR continued to be an independent prognostic factor for all endpoints: OS (HR = 2.38, 95% CI = 1.32–4.28; *p* = 0.003), RFS (HR = 2.37, 95% CI = 1.13–4.99; *p* = 0.020), and CSS (HR = 3.45, 95% CI = 1.56–8.52; *p* = 0.001). Pathological T stage remained a strong independent predictor of OS (HR = 3.50, 95% CI = 1.74–7.01; *p* < 0.001), RFS (HR = 5.12, 95% CI = 2.16–12.2; *p* < 0.001), and CSS (HR = 12.5, 95% CI = 3.77–41.7; *p* < 0.001). LVI was independently associated with OS (HR = 1.99, 95% CI = 1.01–3.92; *p* = 0.043), whereas its association with RFS (*p* = 0.081) and CSS (*p* = 0.170) was not statistically significant after adjustment.

## 4. Discussion

In this retrospective cohort of patients undergoing RC for bladder cancer, a high preoperative GLR was significantly associated with inferior OS, RFS, and CSS. Notably, GLR retained independent prognostic value in both a preoperative model and pathology-adjusted model, suggesting that GLR captures prognostic information beyond conventional staging and postoperative pathology. This cross-model consistency is clinically meaningful because it indicates that GLR may be informative at two distinct decision points: (i) before surgery, when treatment planning and counseling must rely on clinical variables, and (ii) after surgery, when definitive pathology becomes available, and postoperative management is refined.

Multiple inflammation- and nutrition-related indices derived from routine blood tests have been investigated as prognostic biomarkers in bladder cancer patients undergoing RC. Systemic inflammatory response markers, including NLR, PLR, and LMR, have been repeatedly associated with outcomes after RC [3], and a systematic review and meta-analysis further supported the prognostic value of preoperative hematologic biomarkers in this setting [4]. Representative RC cohorts have also reported prognostic roles for SII and other composite indices [8,10,11,12,13], and the AGR has been investigated as a nutrition–inflammation marker associated with post-RC outcomes [14]. More recently, Russo et al. applied decision-curve analysis to evaluate the incremental clinical net benefit of adding systemic indices to conventional models (e.g., SII- and SIRI-based frameworks) [15,16]. In our cohort, NLR differed between GLR strata, suggesting partial overlap in their underlying biological domains; however, GLR remained prognostically informative in sensitivity analyses that treated ln(GLR) as a continuous variable and additionally adjusted for NLR (Supplementary Materials Tables S1–S3). Taken together, these findings suggest that GLR is not merely a proxy for a single inflammatory index, but may capture complementary systemic information by integrating a redox-related marker (GGT) with an immune-status marker (absolute lymphocyte count). Moreover, GLR has been associated with adverse outcomes in hepatocellular carcinoma and intrahepatic cholangiocarcinoma, supporting the potential generalizability of this systemic axis [17,18,19]. Systemic indices have also been explored in upper tract urothelial carcinoma, suggesting that such biomarkers may be relevant across urothelial malignancies [20,21].

Variant histology is increasingly recognized as a driver of aggressive disease and can confound biomarker-based risk stratification [22,23]. In our cohort, variant histology was more frequent in the high-GLR group. We therefore interpret the association between GLR and outcomes with caution. Importantly, GLR remained prognostic in models adjusted for key clinical and pathological factors, and its signal persisted in sensitivity analyses using ln(GLR) as a continuous variable and after additional adjustment for NLR and surgery year (Tables S1–S4). Nevertheless, because of the limited number of events and the need to avoid model overfitting, residual confounding by variant histology cannot be fully excluded. Future multicenter studies with larger event counts should evaluate the incremental value of GLR with explicit adjustment for variant histology and within histologic subtypes.

Why GLR may add value beyond GGT alone deserves emphasis. Experimental studies suggest that GGT-related activity within the tumor microenvironment may facilitate oxidative stress adaptation and resistance to therapy [24]. Serum GGT as a single biomarker can be influenced by non-cancer conditions, whereas GLR incorporates the lymphocyte count, embedding an immune-status dimension into a GGT-based signal. Prior studies have shown prognostic associations for serum GGT in RC cohorts and in advanced urothelial carcinoma [7,25,26]. Our results demonstrate that the composite GLR remained prognostic after adjustment for key clinical and pathological covariates, supporting the potential clinical utility of integrating host immunity into a GGT-based risk assessment. From a clinical standpoint, the association between preoperative GLR and RFS is especially relevant because RFS more directly reflects early relapse dynamics compared with OS and CSS, which can be affected by competing risks and subsequent therapies.

From a clinical standpoint, a readily available preoperative biomarker that refines risk stratification could support more concrete, risk-adapted decision-making. Given the substantial global burden of bladder cancer [27], inexpensive markers that refine risk stratification remain clinically relevant. For example, patients with high GLR could be prioritized for intensified postoperative surveillance (earlier cross-sectional imaging, shorter follow-up intervals), careful counseling regarding early relapse risk, and consideration for clinical trial enrollment when available. In settings where adjuvant systemic therapy is considered after RC, GLR may complement pathological risk factors by identifying patients whose systemic milieu suggests higher relapse risk. Importantly, these potential applications should be viewed as hypothesis-generating; prospective studies are required to determine whether GLR-guided strategies improve outcomes, and whether GLR adds incremental predictive performance beyond established indices (e.g., NLR/SII) in head-to-head comparisons [8,10,11,12,13,14].

This study has some limitations. Given the relatively limited number of events (53 deaths) and covariates in the multivariable model, the events-per-variable (EPV) was approximately 4.4 (53/12), and the findings should be interpreted with caution regarding potential overfitting. It was retrospective and single-center, and residual confounding is possible despite multivariable adjustment. The GLR cut-off was defined by the cohort median, which is appropriate for an exploratory analysis but may not represent the optimal threshold for broader clinical use; accordingly, we complemented the dichotomized analysis with continuous ln(GLR) sensitivity analyses (Supplementary Materials Tables S1–S4). Because the number of events was modest relative to the number of covariates (e.g., OS deaths = 53 in the full cohort), overfitting remains a concern and supports the need for external validation. Serum GGT is susceptible to non-malignant influences, and treatment strategies evolved over the long study period; we therefore additionally adjusted for surgery year as a proxy for temporal changes (Supplementary Materials Table S4). Finally, CSS was analyzed using cause-specific Cox models with non-cancer deaths censored; competing-risk approaches may provide complementary insight and should be explored in larger cohorts. In addition, we dichotomized T stage for parsimony; sensitivity analyses using ordinal staging may provide complementary insight and should be considered in larger cohorts with sufficient events.

## 5. Conclusions

In conclusion, preoperative GLR was independently associated with OS, RFS, and CSS in patients undergoing RC for bladder cancer, and this association remained robust across both preoperative and pathology-adjusted models. Given its simplicity and low cost, GLR may represent a practical adjunct to conventional clinicopathological risk stratification and merits prospective multicenter validation and integration into risk-adapted postoperative management strategies.

## Figures and Tables

**Table 1:** Baseline clinicopathological characteristics according to preoperative GLR.

Parameters	Entire	Low GLR	High GLR	*p*-Value
Number of patients	110	54	56	-
Age (years)	72 [65–77]	72 [65–77]	71.5 [65–77]	0.822
Sex (men/women)	85/25	38/16	47/6	0.090
BMI (kg/m^2^)	23.9 [20.6–25.0]	22.8 [20.5–25.0]	23.6 [20.6–25.0]	0.488
Smoking history				0.431
No (%)	49 (44.5)	22 (40.7)	27 (48.2)	
Yes (%)	61 (55.5)	32 (59.3)	29 (51.8)	
ECOG-PS				0.536
≤2 (%)	94 (85.5)	45 (83.3)	49 (87.5)	
>2 (%)	16 (14.5)	9 (16.7)	7 (12.5)	
Laboratory examination				
AST (U/L)	19.0 [15.8–23.3]	17.0 [15.0–21.0]	21.5 [16.0–25.0]	0.078
ALT (U/L)	16.0 [12.8–21.3]	14.5 [11.0–19.0]	16.0 [13.0–25.8]	0.086
ALP (U/L)	202.5 [127.0–274.0]	198 [103.8–273.0]	219 [145.3–277.8]	0.499
CRP (mg/dL)	0.15 [0.05–0.45]	0.11 [0.05–0.28]	0.18 [0.05–0.88]	0.101
GGT (U/L)	26.0 [19.0–40.3]	19.5 [15.0–23.3]	38.0 [31.3–64.8]	<0.001
Neutrophil count (×10^9^/L)	4.10 [2.99–5.66]	3.92 [2.90–5.27]	4.23 [3.11–6.16]	0.226
Platelet count (×10^9^/L)	221.5 [187.5–286.0]	221.0 [186.3–287.5]	225.0 [186.5–279.0]	0.806
Lymphocyte count (×10^9^/L)	1.58 [1.23–2.13]	1.79 [1.31–2.27]	1.39 [1.19–2.04]	0.036
GLR	17.0 [12.4–26.1]	14.5 [9.38–22.1]	32.5 [21.1–57.4]	<0.001
NLR	2.49 [1.71–4.00]	2.25 [1.63–3.17]	3.18 [1.72–4.58]	0.036
PLR	142.7 [103.6–204.8]	132.6 [102.8–189.7]	150.6 [104.4–218.3]	0.173
Treatment				
Neoadjuvant chemotherapy (%)	65 (59.0)	29 (53.7)	36 (64.3)	0.259
Adjuvant chemotherapy (%)	12 (10.9)	6 (11.1)	6 (10.7)	0.947
Operation				0.549
Open surgery	18 (16.4)	10 (18.5)	8 (14.3)	
Laparoscopic/robotic surgery	92 (83.6)	44 (81.5)	48 (85.7)	
Urinary diversion				0.187
Ileal conduit	77 (70.0)	34 (63.0)	43 (76.8)	
Cutaneous ureterostomy	12 (10.9)	6 (11.1)	6 (10.7)	
Orthotopic neobladder	21 (19.1)	14 (25.9)	7 (12.5)	
Clinicopathological features				
cT stage				0.697
1 (%)	17 (15.5)	10 (18.5)	7 (12.5)	
2 (%)	41 (37.2)	21 (38.9)	20 (35.7)	
3 (%)	39 (35.5)	18 (33.3)	21 (37.5)	
4 (%)	13 (11.8)	5 (9.3)	8 (14.3)	
Lymph node metastasis (cN)				0.072
Negative (%)	101 (91.8)	47 (87.0)	54 (96.4)	
Positive (%)	9 (8.2)	7 (13.0)	2 (3.6)	
Pathological stage				0.588
pT0 (%)	17 (15.5)	11 (20.4)	6 (10.7)	
pT1 (%)	37 (33.6)	19 (35.2)	18 (32.1)	
pT2 (%)	15 (13.6)	7 (13.0)	8 (14.3)	
pT3 (%)	30 (27.2)	12 (22.2)	18 (32.1)	
pT4 (%)	11 (10.0)	5 (9.3)	6 (10.7)	
Concomitant CIS				0.741
Negative (%)	83 (75.5)	40 (74.1)	43 (76.8)	
Positive (%)	27 (24.5)	14 (25.9)	13 (23.2)	
Histopathological classification				0.038
UC (%)	77 (70.0)	43 (79.6)	34 (60.7)	
UC with variant histology (%)	33 (30.0)	11 (20.4)	22 (39.3)	
Lymphovascular invasion				0.236
Negative (%)	63 (57.3)	34 (63.0)	29 (51.8)	
Positive (%)	47 (42.7)	20 (37.0)	27 (48.2)	
Tumor grade				0.660
Low (%)	7 (6.4)	4 (7.4)	3 (5.4)	
High (%)	103 (93.6)	50 (92.6)	53 (94.6)	

Data are presented as median [interquartile range] or *n* (%). *p*-values were calculated using the Mann–Whitney U-test for continuous variables and the chi-square test or Fisher’s exact test for categorical variables, as appropriate. Abbreviations: GLR, gamma-glutamyltransferase-to-lymphocyte ratio; GGT, gamma-glutamyltransferase; BMI, body mass index; ECOG-PS, Eastern Cooperative Oncology Group performance status; AST, aspartate aminotransferase; ALT, alanine aminotransferase; ALP, alkaline phosphatase; CRP, C-reactive protein; NLR, neutrophil-to-lymphocyte ratio; PLR, platelet-to-lymphocyte ratio; CIS, carcinoma in situ; UC, urothelial carcinoma; cT, clinical T stage; cN, clinical nodal status.

**Table 2:** Univariate and multivariate Cox proportional hazards analyses for OS, RFS, and CSS using a preoperative model (Model 1).

Variable	Univariate Analysis			Multivariate Analysis		
	HR	95% CI	*p*-Value	HR	95% CI	*p*-Value
OS						
Sex (male vs. female)	1.58	0.85–2.92	0.149	-	-	-
Age	1.06	1.03–1.10	<0.001	1.05	1.01–1.09	0.006
ECOG-PS (>2 vs. ≤2)	2.21	1.15–4.23	0.017	1.37	0.66–2.84	0.403
Smoking history (yes vs. no)	2.02	1.17–3.49	0.012	1.66	0.94–2.94	0.081
cT stage (≥3 vs. ≤2)	2.06	1.18–3.59	0.011	1.62	0.89–2.95	0.117
cN (cN+ vs. cN0)	1.21	0.43–3.36	0.717	-	-	-
Neoadjuvant chemotherapy (yes vs. no)	1.16	0.67–2.01	0.605	-	-	-
Operation (open vs. laparoscopic/robotic)	2.06	0.89–4.76	0.092	-	-	-
C-reactive protein	1.21	0.99–1.40	0.058	-	-	-
GLR (>17.0 vs. ≤17.0)	1.91	1.10–3.32	0.022	2.23	1.25–3.98	0.006
RFS						
Sex (male vs. female)	1.09	0.47–2.52	0.840	-	-	-
Age	1.03	0.99–1.08	0.100	-	-	-
ECOG-PS (>2 vs. ≤2)	1.80	0.78–4.16	0.196	-	-	-
Smoking history (yes vs. no)	2.25	1.11–4.57	0.024	2.27	1.12–4.61	0.024
cT stage (≥3 vs. ≤2)	2.04	1.01–4.14	0.048	2.00	1.01–4.08	0.048
cN (cN+ vs. cN0)	1.14	0.35–3.75	0.809	-	-	-
Neoadjuvant chemotherapy (yes vs. no)	1.50	0.75–3.01	0.253	-	-	-
Operation (open vs. laparoscopic/robotic)	1.53	0.68–3.43	0.301	-	-	-
C-reactive protein	0.97	0.59–1.25	0.847	-	-	-
GLR (>17.0 vs. ≤17.0)	2.07	1.01–4.24	0.047	2.02	1.01–4.16	0.045
CSS						
Sex (male vs. female)	1.14	0.46–2.81	0.779	-	-	-
Age	1.05	1.01–1.10	0.020	1.04	0.997–1.10	0.068
ECOG-PS (>2 vs. ≤2)	2.30	0.98–5.43	0.057	-	-	-
Smoking history (yes vs. no)	2.20	1.05–4.62	0.037	1.82	0.84–3.92	0.125
cT stage (≥3 vs. ≤2)	3.18	1.43–7.04	0.004	2.54	1.12–5.76	0.021
cN (cN+ vs. cN0)	1.25	0.38–4.17	0.719	-	-	-
Neoadjuvant chemotherapy (yes vs. no)	1.04	0.50–2.20	0.908	-	-	-
Operation (open vs. laparoscopic/robotic)	1.34	0.48–3.70	0.578	-	-	-
C-reactive protein	1.13	0.78–1.44	0.410	-	-	-
GLR (>17.0 vs. ≤17.0)	3.19	1.41–7.24	0.005	3.31	1.43–7.66	0.003

Model 1 included only variables available before surgery. Variables entered into each multivariate model are shown in the table. Hazard ratios (HRs) are presented with 95% confidence intervals (CIs). *p* values < 0.05 were considered statistically significant. Abbreviations: HR, hazard ratio; CI, confidence interval; ECOG-PS, Eastern Cooperative Oncology Group performance status; GLR, gamma-glutamyltransferase-to-lymphocyte ratio; OS, overall survival; RFS, recurrence-free survival; CSS, cancer-specific survival; cN, clinical nodal status.

**Table 3:** Univariate and multivariate Cox proportional hazards analyses for OS, RFS, and CSS using a postoperative model (Model 2).

Variable	Univariate Analysis			Multivariate Analysis		
	HR	95% CI	*p*-Value	HR	95% CI	*p*-Value
OS						
Sex (male vs. female)	1.58	0.85–2.92	0.149	-	-	-
Age	1.06	1.03–1.10	<0.001	1.03	0.99–1.08	0.079
ECOG-PS (>2 vs. ≤2)	2.21	1.15–4.23	0.017	1.41	0.71–2.81	0.336
Histology (with vs. without variant histology)	1.62	0.92–2.85	0.093	-	-	-
pT stage (≥3 vs. ≤2)	5.98	3.29–10.9	<0.001	3.50	1.74–7.01	<0.001
LVI (positive vs. negative)	4.05	2.29–7.15	<0.001	1.99	1.01–3.92	0.043
C-reactive protein	1.21	0.99–1.40	0.058	-	-	-
GLR (>17.0 vs. ≤17.0)	1.91	1.10–3.32	0.022	2.38	1.32–4.28	0.003
RFS						
Sex (male vs. female)	1.09	0.47–2.52	0.840	-	-	-
Age	1.03	0.99–1.08	0.100	-	-	-
ECOG-PS (>2 vs. ≤2)	1.80	0.78–4.16	0.196	-	-	-
Histology (with vs. without variant histology)	1.24	0.59–2.63	0.576	-	-	-
pT stage (≥3 vs. ≤2)	7.12	3.33–15.2	<0.001	5.12	2.16–12.2	<0.001
LVI (positive vs. negative)	4.09	1.96–8.53	<0.001	2.11	0.90–4.97	0.081
C-reactive protein	0.97	0.59–1.25	0.847	-	-	-
GLR (>17.0 vs. ≤17.0)	2.07	1.01–4.24	0.047	2.37	1.13–4.99	0.020
CSS						
Sex (male vs. female)	1.14	0.46–2.81	0.779	-	-	-
Age	1.05	1.01–1.10	0.020	1.02	0.97–1.07	0.533
ECOG-PS (>2 vs. ≤2)	2.30	0.98–5.43	0.057	-	-	-
Histology (with vs. without variant histology)	1.81	0.85–3.83	0.124	-	-	-
pT stage (≥3 vs. ≤2)	18.4	6.28–53.8	<0.001	12.5	3.77–41.7	<0.001
LVI (positive vs. negative)	5.72	2.52–13.0	<0.001	1.90	0.73–4.93	0.170
C-reactive protein	1.13	0.78–1.44	0.410	-	-	-
GLR (>17.0 vs. ≤17.0)	3.19	1.41–7.24	0.005	3.45	1.56–8.52	0.001

Model 2 additionally incorporated postoperative pathological variables. Variables entered into each multivariate model are shown in the table. Hazard ratios (HRs) are presented with 95% confidence intervals (CIs). *p* values < 0.05 were considered statistically significant. Abbreviations: HR, hazard ratio; CI, confidence interval; pT, pathological T stage; LVI, lymphovascular invasion; ECOG-PS, Eastern Cooperative Oncology Group performance status; GLR, gamma-glutamyltransferase-to-lymphocyte ratio; OS, overall survival; RFS, recurrence-free survival; CSS, cancer-specific survival.

## Data Availability

The data supporting this study are available from the corresponding author upon reasonable request. The data are not publicly accessible due to privacy and ethical considerations.

## Supplementary Materials

The following supporting information can be downloaded at: https://www.mdpi.com/article/10.3390/medicina62020343/s1, Table S1: Univariable and multivariable Cox regression analyses of ln(GLR) as a continuous variable (preoperative model); Table S2: Univariable and multivariable Cox regression analyses of NLR as a continuous variable (preoperative model); Table S3: Multivariable Cox regression analyses including ln(GLR) and NLR (preoperative model); Table S4: Multivariable Cox regression analyses including ln(GLR), NLR, and surgery year (preoperative model).

## Abbreviations

The following abbreviations are used in this manuscript:

AGR	Albumin–Globulin Ratio
ALP	Alkaline Phosphatase
ALT	Alanine Aminotransferase
AST	Aspartate Aminotransferase
BMI	Body Mass Index
CI	Confidence Interval
CIS	Carcinoma In Situ
CRP	C-Reactive Protein
CSS	Cancer-Specific Survival
cN	Clinical Nodal Status
cT	Clinical T Stage
ECOG-PS	Eastern Cooperative Oncology Group Performance Status
GGT	Gamma-Glutamyltransferase
GLR	Gamma-Glutamyltransferase-to-Lymphocyte Ratio
HR	Hazard Ratio
IQR	Interquartile Range
LMR	Lymphocyte-to-Monocyte Ratio
LVI	Lymphovascular Invasion
MIBC	Muscle-Invasive Bladder Cancer
NLR	Neutrophil-to-Lymphocyte Ratio
OS	Overall Survival
PLR	Platelet-to-Lymphocyte Ratio
RC	Radical Cystectomy
RFS	Recurrence-Free Survival
SII	Systemic Immune-Inflammation Index
