# Supplementary material for: Preoperative Gamma-Glutamyltransferase-to-Lymphocyte Ratio as an Independent Prognostic Biomarker in Patients Undergoing Radical Cystectomy for Bladder Cancer
Source: Medicina (Kaunas). 2026 Feb 8;62(2):343. doi: 10.3390/medicina62020343 (PMC12942478; doi:10.3390/medicina62020343)
Supplement: Supplementary file 1 [file medicina-62-00343-s001.zip › medicina-4106299-supplementary.pdf]

Table S1. Univariable and multivariable Cox regression analyses of ln(GLR) as a continuous variable (preoperative model).

Variable	Univariate analysis			Multivariate analysis		
	**HR**	**95% CI**	***P*-value**	**HR**	**95% CI**	***P*-value**
Overall survival						
ln(GLR)	1.64	1.10-2.37	0.011	1.55	1.07-2.25	0.020
Recurrence-free survival						
ln(GLR)	1.71	1.04-2.65	0.025	1.51	1.05-2.15	0.020
Cancer-specific survival						
ln(GLR)	1.84	1.10-2.92	0.014	1.62	1.01-2.53	0.016

Footnote: The multivariable model was adjusted for the same preoperative covariates as in Table 2; for brevity, only ln(GLR) is shown. Univariable results for the other preoperative covariates are identical to those shown in Table 2 and are therefore not repeated here. ln(GLR) indicates the natural-log–transformed GLR. HRs are presented with 95% CIs. *P* values <0.05 were considered statistically significant. Abbreviations: HR, hazard ratio; CI, confidence interval; GLR, gamma-glutamyltransferase-to-lymphocyte ratio.

Table S2. Univariable and multivariable Cox regression analyses of NLR as a continuous variable (preoperative model).

Variable	Univariate analysis			Multivariate analysis		
	**HR**	**95% CI**	***P*-value**	**HR**	**95% CI**	***P*-value**
Overall survival						
NLR	1.18	1.09-1.28	<0.001	1.16	1.06-1.26	<0.001
Recurrence-free survival						
NLR	1.07	0.93-1.16	0.208	-	-	-
Cancer-specific survival						
NLR	1.18	1.00-1.35	0.027	1.14	0.94-1.34	0.150

Footnote: The multivariable model was adjusted for the same preoperative covariates as in Table 2; for brevity, only NLR is shown. Univariable results for the other preoperative covariates are identical to those shown in Table 2 and are therefore not repeated here. Multivariable analysis for recurrence-free survival was not performed because NLR was not significant in univariable analysis. HRs are presented with 95% CIs. *P* values <0.05 were considered statistically significant. Abbreviations: HR, hazard ratio; CI, confidence interval; NLR, neutrophil-to-lymphocyte ratio.

Table S3. Multivariable Cox regression analyses including ln(GLR) and NLR as continuous variables (preoperative model).

Variable	Multivariate analysis		
	**HR**	**95% CI**	***P*-value**
Overall survival			
ln(GLR)	1.12	1.01-1.22	0.025
NLR	1.14	1.04-1.25	0.005
Recurrence-free survival			
ln(GLR)	1.39	0.83-2.26	0.187
NLR	1.02	0.86-1.13	0.804
Cancer-specific survival			
ln(GLR)	1.47	0.89-2.43	0.128
NLR	1.11	0.92-1.33	0.274

Footnote: The multivariable model was adjusted for the same preoperative covariates as in Table 2; for brevity, only ln(GLR) and NLR are shown. Univariable results for the other preoperative covariates are identical to those shown in Table 2 and are therefore not repeated here. HRs are presented with 95% CIs. *P* values <0.05 were considered statistically significant. Abbreviations: HR, hazard ratio; CI, confidence interval; GLR, gamma-glutamyltransferase-to-lymphocyte ratio; NLR, neutrophil-to-lymphocyte ratio.

Table S4. Multivariable Cox regression analysis for overall survival including ln(GLR), NLR, and surgery year as continuous variables (preoperative model).

Variable	Multivariate analysis		
	**HR**	**95% CI**	***P*-value**
Overall survival			
ln(GLR)	1.34	1.02-1.99	0.020
NLR	1.13	1.03-1.23	0.009
Surgery year (radical cystectomy, continuous)	1.04	0.94-1.14	0.467

Footnote: The multivariable model was additionally adjusted for the same preoperative covariates as in Table 2; for brevity, only ln(GLR), NLR, and surgery year are shown. HRs are presented with 95% CIs. P values <0.05 were considered statistically significant.

Abbreviations: HR, hazard ratio; CI, confidence interval; GLR, gamma-glutamyltransferase-to-lymphocyte ratio; NLR, neutrophil-to-lymphocyte ratio.
